# Tea Polyphenols in the COVID-19 Era: Mechanistic Insights and Translational Challenges

**DOI:** 10.3390/cimb48040379

**Published:** 2026-04-05

**Authors:** Harrison Chang, Chi-Sheng Wu, Ting-Yu Yeh, Wen-Chin Ko

**Affiliations:** 1 Institute of Marine and Environmental Technology, University of Maryland Baltimore County, Baltimore, MD 21202, USA; s14328@kcis.com.tw (H.C.); yehty@auxergen.com (T.-Y.Y.); 2 Agricultural Biotechnology Laboratory, Auxergen Inc., Riti Rossi Colwell Center, Baltimore, MD 21202, USA; 3 Renal Care Research and Health Promotion Association, New Taipei City 220050, Taiwan; luke.chisheng@gmail.com; 4 Division of Cardiac Electrophysiology, Department of Cardiovascular Center, Cathay General Hospital, Taipei 106438, Taiwan; 5 School of Medicine, Fu Jen Catholic University, New Taipei City 242062, Taiwan

**Keywords:** polyphenols, nutraceuticals, COVID-19

## Abstract

The severe acute respiratory syndrome coronavirus 2 (SARS-CoV-2) has driven the global COVID-19 pandemic, imposing a tremendous burden on public health. As the virus continually evolves through rapid mutations, the pandemic has transitioned into a prolonged endemic phase. Despite the development of novel drugs and vaccines, clinical outcomes remain suboptimal for vulnerable populations, including the elderly and those with comorbidities or compromised immunity. Tea polyphenols, a class of structurally diverse and bioactive nutraceuticals, may modulate viral entry, replication, and host inflammatory pathways implicated in disease progression through pleiotropic effects on viral attachment, membrane fusion, intracellular replication, and proteolytic processing. Here, we provide an updated chemo-biological perspective on the antiviral and immunomodulatory mechanisms of tea polyphenols against SARS-CoV-2. Current evidence highlights their potential to serve as promising candidates for further mechanistic and translational investigation as adjunctive strategies and nutraceuticals for COVID-19 management. Importantly, no large-scale randomized controlled trials have yet demonstrated clinical benefit of tea polyphenols in COVID-19.

## 1. Introduction

Coronavirus Disease 2019 (COVID-19), caused by the highly transmissible severe acute respiratory syndrome coronavirus 2 (SARS-CoV-2), has triggered a devastating global pandemic characterized by severe and fatal pneumonia [1]. Historically, the first major wave of a zoonotic coronavirus illness (SARS-CoV-1) emerged in southern China between 2002 and 2004 [1,2]. While the overall mortality rate for SARS-CoV-1 infections was approximately 9%, it approached 50% among individuals over 60 years of age [3]. Subsequently, in 2012, the Middle East respiratory syndrome (MERS) emerged, causing severe pneumonia and multiorgan failure [1]. MERS exhibited an estimated mortality rate of 35%, though this figure may be artificially inflated due to surveillance systems failing to capture mild cases [4]. Emerging in late 2019, COVID-19 rapidly disseminated worldwide, vastly surpassing both SARS and MERS in cumulative infections and geographic distribution. Consequently, the COVID-19 pandemic continues to inflict a tremendous burden on global public health infrastructure [5].

Although the rapid development of antivirals, immunomodulators, and multiple vaccine generations has transformed COVID-19 management, critical therapeutic gaps remain. Existing therapies primarily focus on halting viral replication or mitigating hyperinflammation; however, their efficacy is frequently compromised in high-risk groups. Consequently, older adults, patients with multimorbidity, and the immunocompromised continue to suffer a disproportionately high burden of severe disease and mortality [6,7,8]. In addition, SARS-CoV-2 continues to evolve, and emerging variants may partially evade vaccine-induced and infection-induced immunity, further challenging disease control and therapeutic effectiveness [9]. Breakthrough infections and persistent post-acute sequelae of SARS-CoV-2 infection (PASC, long COVID) also highlight gaps in long-term management, as no definitive treatments are currently available to prevent or reverse chronic inflammation, multiorgan dysfunction, or prolonged symptom burden [10,11]. These limitations underscore the urgent need to explore adjunctive therapeutics and safe, broadly accessible bioactive compounds that may interact with biological pathways relevant to disease progression and could complement existing interventions pending further validation for vulnerable populations.

The structural diversity, stability, safety, and excellent biological activity of tea polyphenols have drawn the attention of researchers, further highlighting their potential as adjunctive therapies against infectious diseases, including COVID-19. Among the hundreds of polyphenolic constituents identified in tea, catechins such as epigallocatechin-3-gallate (EGCG) exhibit multifaceted antiviral activities, ranging from interference with viral receptor binding and membrane fusion to inhibition of viral proteases and replication enzymes [12,13]. In addition to direct antiviral effects, tea polyphenols exert potent immunomodulatory, antioxidant, and anti-inflammatory functions, which may counteract pathological processes implicated in severe COVID-19, such as cytokine dysregulation, oxidative stress, and endothelial injury [14,15,16].

Furthermore, tea is one of the most widely consumed beverages globally, offering significant advantages to vulnerable groups with limited treatment options, thus endowing it with unique application potential and accessibility. Although mechanistic and preclinical evidence is rapidly accumulating, the antiviral and immunomodulatory mechanisms of tea polyphenols against SARS-CoV-2 are not yet fully elucidated. These factors collectively highlight the necessity and timeliness of this focused review to clarify its biochemical mechanisms, therapeutic significance, and future research directions.

This review aims to integrate mechanistic, preclinical, and emerging translational evidence to clarify how tea polyphenols may modulate key stages of the SARS-CoV-2 life cycle, host antiviral responses, and COVID-19 pathophysiology, and to highlight research gaps that warrant further investigation.

## 2. Background of Tea Polyphenols

### 2.1. Chemical Structure Classification of Tea Polyphenols

Tea polyphenols comprise a broad collection of plant-derived secondary metabolites, many of which fall within the flavonoid superfamily. This group includes flavan-3-ols (catechins), theaflavins, thearubigins, and various flavonols. Although structurally related, these compounds differ in the biosynthetic steps and oxidative transformations that give rise to their individual chemical features.

Catechins (Green Tea Polyphenols)

Catechins such as EGCG, EGC, ECG, and EC share the characteristic C6–C3–C6 flavan-3-ol framework but differ in the number and placement of hydroxyl groups as well as the presence or absence of a gallate moiety at the 3-position. These monomeric forms are the predominant polyphenols found in unfermented green tea.

Theaflavins and Thearubigins (Oxidized Polyphenols in Oolong/Black Tea)

During the oxidation step of tea processing, often referred to as fermentation, catechins can react with one another to produce a range of larger phenolic compounds. Among these are the theaflavins, which contain a distinctive benzotropolone core, and the thearubigins, a more complex and variable collection of high-molecular-weight polymers. These oxidation products contribute substantially to the typical color and astringent character associated with black tea.

Flavonols and Other Minor Polyphenols

Tea leaves contain several flavonol aglycones—quercetin, kaempferol, and myricetin are among the more common examples—and these compounds frequently occur together with their corresponding glycosides. The brewed infusion also provides phenolic acids, such as gallic acid. Taken together, these constituents add measurably to the antioxidant profile of tea and help explain its capacity to interact with, and bind, metal ions.

**Figure 1:**
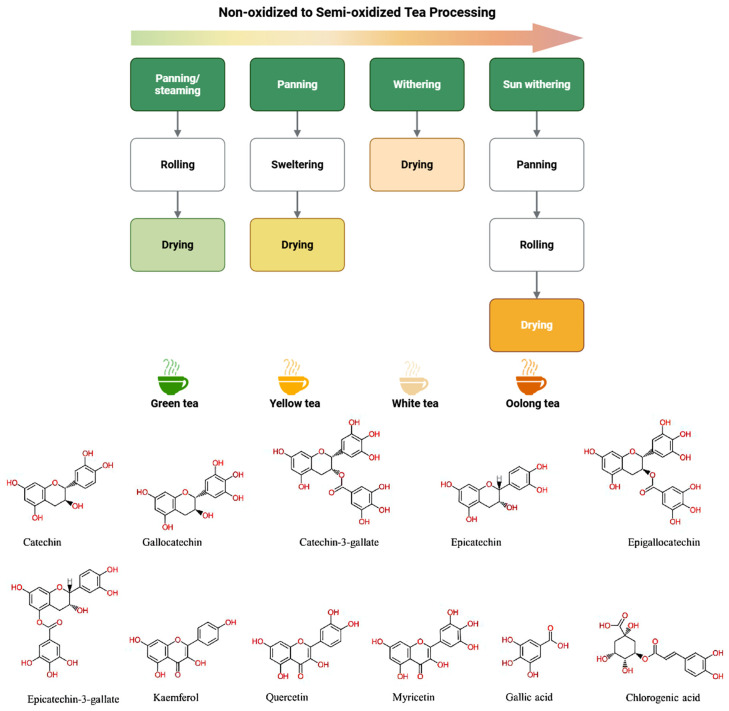
Overview of tea processing and representative polyphenolic constituents. The upper panel depicts the enzymatic oxidation stages determining tea classification (non-fermented, semi-fermented, and fully fermented). The lower panel presents the chemical structures of major monomeric catechins, flavonols, and phenolic acids identified in Camellia sinensis. Structural depictions are adapted from Shaukat et al. (2023) [17], Tea polyphenols: extraction techniques and its potency as a nutraceutical, and are reproduced under the terms of the CC BY 4.0 license. The upper schematic was created with BioRender (Created in BioRender. Harrison Chang and Chi-Sheng Wu (2025) https://www.biorender.com/ accessed on 1 December 2025) under an active academic license.

As illustrated in Figure 1, the polyphenolic profile of tea is primarily determined by the degree of enzymatic oxidation during processing. In non-fermented green tea, the constituents remain predominantly as monomeric catechins, such as EGCG, which accounts for 50–80% of the total catechin content. Conversely, during the production of oolong (semi-fermented) and black tea (fully fermented), these catechins undergo oxidative polymerization mediated by polyphenol oxidase and peroxidase. This process transforms simple catechins into complex, high-molecular-weight oligomers, including theaflavins and thearubigins, which contribute to the characteristic color and astringency of fermented teas. Beyond catechins, the tea leaf matrix contains a diverse array of phenolic compounds, including flavonols (e.g., quercetin and kaempferol) and phenolic acids (e.g., gallic acid and chlorogenic acid), each characterized by distinct aromatic ring substitutions and hydroxyl group configurations that underpin their radical-scavenging and metal-chelating properties.

### 2.2. Biological Activities of Tea Polyphenols

Tea polyphenols exhibit a broad range of biological activities stemming from their redox chemistry, metal-binding capacity, and interactions with cellular signaling pathways Tea polyphenols act through several distinct biochemical features. Their redox activity, their capacity to complex with metal ions, and their interactions with intracellular signaling networks each contribute to the range of biological effects attributed to these compounds.

Antioxidant Properties

Catechins and theaflavins can neutralize a variety of reactive oxygen species (ROS), and a number of experimental reports indicate that they assist cells in keeping oxidative pressure in check by reinforcing intrinsic antioxidant systems. A notable example is their capacity to stimulate components of the Nrf2 network, which regulates many of the genes responsible for oxidative stress protection [14].

Anti-inflammatory and Immunomodulatory Effects

Reports in the literature describe that tea polyphenols can diminish the release of several inflammatory cytokines (particularly IL-6 and TNF-α) and may also blunt NF-κB activity while steering macrophages toward phenotypes associated with lower inflammatory output [18]. These effects are clinically relevant because, in severe respiratory infections, persistent and dysregulated inflammatory signaling is a major contributor to tissue damage and clinical decline.

Antiviral Activity

EGCG, theaflavins, and flavonols have demonstrated broad-spectrum antiviral functions by:

- interfering with viral attachment and membrane fusion [19];
- inhibiting viral proteases (e.g., SARS-CoV-2 3CLpro) [13];
- disrupting viral RNA polymerase activity [20].

These activities provide a mechanistic foundation for examining tea polyphenols as adjunctive nutraceuticals against respiratory viruses.

### 2.3. Relationship Between Tea Polyphenols, Respiratory Diseases, and Viral Infections (Rewritten Version)

Recent studies suggest that tea polyphenols contribute to respiratory health through a combination of antioxidant, anti-inflammatory, and antiviral mechanisms. Beyond their established redox-modulating properties, accumulating experimental evidence indicates that these compounds can stabilize airway epithelial integrity and modulate host immune responses in the respiratory tract. Moreover, tea-derived polyphenols have been reported to interfere with multiple stages of viral infection, including viral entry, replication, and host–virus interactions. Collectively, these findings support the potential role of tea polyphenols in reducing susceptibility to respiratory infections and modulating disease progression.

Respiratory Protection through Antioxidant and Anti-inflammatory Pathways

Tea polyphenols, particularly catechins such as EGCG, exert potent antioxidant and anti-inflammatory effects that are highly relevant in pulmonary tissues. Excessive production of ROS contributes to epithelial dysfunction and inflammatory damage in the respiratory system. Experimental studies in both acute and chronic lung injury models have demonstrated that EGCG can attenuate oxidative stress and suppress pro-inflammatory signaling pathways, including NF-κB activation and NLRP3 inflammasome activation. These effects are accompanied by reduced production of inflammatory cytokines and improved epithelial barrier integrity, ultimately contributing to decreased inflammation in bronchial and alveolar compartments [21,22].

Antiviral Effects Against Human Coronaviruses and Influenza Viruses

Tea catechins have demonstrated broad-spectrum antiviral activity against multiple respiratory viruses. EGCG has been shown to inhibit the replication of human coronaviruses (e.g., OC43 and 229E) as well as influenza A and B viruses. Mechanistically, EGCG can disrupt viral envelope integrity, interfere with spike protein–receptor interactions, and inhibit endosomal membrane fusion required for viral entry. These multi-target antiviral actions suggest that tea polyphenols may function as natural virucidal agents and hold potential as preventive or adjunctive strategies, although robust clinical evidence remains limited [13,19].

Relevance to SARS-CoV-2 Pathogenesis

Emerging evidence indicates that tea polyphenols target key molecular processes involved in SARS-CoV-2 infection. EGCG and theaflavins have been reported to inhibit viral enzymes, including the main protease (3CLpro/Mpro) and RNA-dependent RNA polymerase (RdRp), thereby suppressing viral replication in vitro. In addition, their immunomodulatory effects—such as regulation of interferon signaling and attenuation of hyperinflammatory responses—highlight their potential translational relevance as nutraceutical or adjunctive agents in COVID-19 management [23,24,25].

## 3. SARS-CoV-2 Pathogenesis and Infection Mechanisms

Focusing on the main infection cycle of SARS-CoV-2 can guide efforts in medicinal chemistry to discover new drug targets for this devastating disease. SARS-CoV-2 infection is initiated by the interaction between the viral spike (S) protein and the host receptor angiotensin-converting enzyme 2 (ACE2) [26]. After ACE2 binding, the host transmembrane serine protease 2 (TMPRSS2) is required for S protein cleavage to enhance viral entry and activate furin-cleavable fusion proteins prior to fusion to the host cell membrane [27,28].

In addition to therapies with immunomodulatory effects on human cells, certain therapeutic strategies against SARS-CoV-2 have been developed from mechanistic insights, including targeting the SARS-CoV-2 life cycle at the levels of binding, entry, replication, protein processing [29]. Nonetheless, the virus constantly mutates and manifests diverse antigenicity, causing standard therapies such as vaccines and monoclonal antibodies to lose their effectiveness. Therefore, natural products modulating ACE2–spike interactions or host antiviral responses possess may provide beneficial effects by enhancing the host immune response [30]. Polyphenols, which possess antioxidant and anti-inflammatory properties, also exhibit broad antiviral activities against diverse groups of viruses, such as influenza virus, hepatitis viruses, herpes simplex virus 1, HIV, and Epstein–Barr virus [31,32]. The present article summarizes *in vitro* and *in silico* research regarding anti-SARS-CoV-2 abilities of polyphenols and discusses their potential to accelerate antiviral drug discovery. This review provides a comprehensive overview of the antiviral mechanisms of polyphenols and discusses their implications for future therapeutic development against SARS-CoV-2.

These mechanistic insights provide a rationale for exploring multi-target agents such as tea polyphenols, which may modulate viral entry, replication, and host inflammatory responses.

## 4. Antiviral Mechanisms of Tea Polyphenols

**Figure 2:**
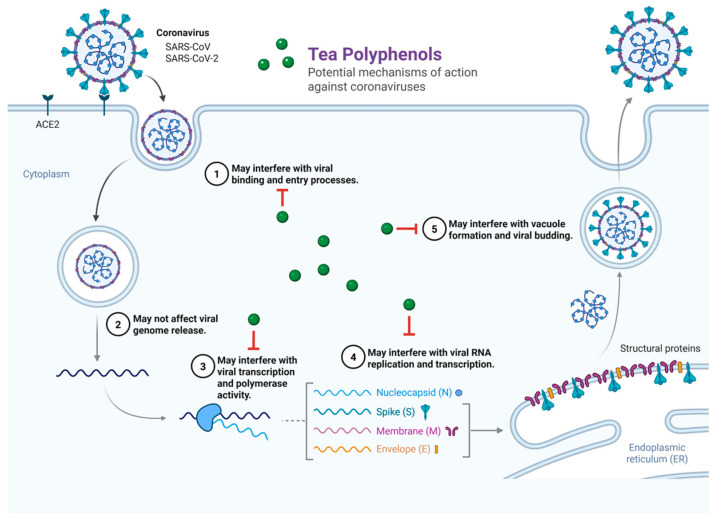
Proposed antiviral targets of tea polyphenols across the coronavirus life cycle. Numerical labels (1–5) denote specific mechanistic checkpoints targeted by tea polyphenols, ranging from initial viral entry to progeny virion maturation. Detailed functional descriptions of each stage are provided in the main text of Section 4. Created with BioRender.com (Created in BioRender. Harrison Chang and Chi-Sheng Wu (2025) https://www.biorender.com/ accessed on 3 December 2025).

The antiviral potential of tea polyphenols is characterized by their ability to modulate coronavirus infection at several distinct mechanistic checkpoints. As illustrated in Figure 2, these pleiotropic actions span multiple stages of the viral life cycle. Specifically, tea-derived compounds may interfere with viral attachment and membrane fusion (1), suppress viral transcription and polymerase activity (3), and restrict RNA replication and protein processing (4). Furthermore, emerging evidence suggests these polyphenols may disrupt late-stage virion maturation and budding processes (5). Conversely, the stage of viral genome release following endosomal uptake (2) appears to be less affected by polyphenolic interference. This multi-targeted approach underscores the therapeutic significance of tea polyphenols as robust adjunctive agents capable of inhibiting viral propagation through diverse biochemical pathways.

### 4.1. Modulation of ACE2 and Viral EntryACE2 Distribution

A myriad of organ/systems contain ACE2, a type I transmembrane metallocarboxypeptidase, especially for lungs and the respiratory system [33]. ACE2-expressing epithelial cells in the nasal cavity and respiratory mucosa facilitate SARS-CoV-2 attachment and entry, leading to host infection. ACE2 is also expressed in cardiovascular, gastrointestinal, and renal tissues, contributing to their susceptibility during systemic infection. Focusing on the entry of SARS-CoV-2 into the lung epithelial cells of hosts, ACE2 could serve as a targeted therapy [34].

Direct inhibition of ACE2–spike binding

From the perspectives of ACE2 binding affinity, several studies reported polyphenols as potential viral entry inhibitors [30]. Resveratrol, a phenolic compound found naturally in fruits, nuts, flowers, seeds and bark of different plants, exhibits a potential to mitigate the severity of SARS-CoV-2 infection [35]. Emerging evidence also reveals tea polyphenols, EGCG, may interfere with viral attachment by binding to ACE2 or the spike receptor-binding domain (RBD) [31]. Although EGCG has been reported to interact with viral proteins such as 3CLpro, its ability to prevent SARS-CoV-2 infection *in vivo* remains to be clarified. Recent *in vitro* and *in silico* studies further suggest that several flavonoids may suppress spike–ACE2 binding. Notably, eriodictyol has been demonstrated to reduce SARS-CoV-2 cellular entry by interfering with spike–ACE2 interaction, supporting the notion that natural flavonoids may modulate early viral attachment events [36].

According to molecular docking models, certain polyphenols have been predicted to interact with ACE2 binding residues through hydrogen bonding [37]. Both catechin-derived compounds and other polyphenols have also been suggested to bind the ACE2–RBD interface of the viral S protein [38]. However, these findings are based on *in silico* predictions, and *in vitro* and *in vivo* validation is required to determine whether such interactions meaningfully influence viral entry.

Regulation of ACE2 abundance and lung protection

Previous SARS research demonstrated that viral infection downregulates ACE2, contributing to acute lung injury. Restoration of ACE2 or use of soluble ACE2 protects against lung damage in mouse models [39]. Likewise, soluble forms of ACE2 therapy as a decoy receptor for the S protein interrupted SARS-CoV and SARS-CoV-2 viral entry and infection in cell models [40]. In light of this, Apeiron Biologics advanced soluble recombinant human ACE2 (APN01) into Phase II clinical trials for COVID-19 [41]. While preliminary reports indicated good safety and modulation of RAS-related biomarkers, peer-reviewed Phase II efficacy data have not been published, and clinical benefit remains uncertain. Although certain non-tea polyphenols have been reported to modulate the renin–angiotensin system by regulating angiotensin II signaling and potentially alleviating lung injury [42,43,44], the present review primarily focuses on tea-derived catechins. Because dietary polyphenols may influence SARS-CoV-2 disease severity by regulating ACE2 expression and function, previous studies have explored the interactions between dietary factors, ACE2 gene variations, and COVID-19 outcomes. For example, supplementation with certain non-tea polyphenols has been reported to increase ACE2 expression in high-fat diet models compared with high-fat diet alone [35]. Collectively, these findings suggest that, beyond direct interference with ACE2–virus binding, tea polyphenols may ameliorate lung injury by modulating ACE2 abundance and activity. In this context, tea-derived catechins, particularly EGCG, may represent a relevant and focused class of compounds for further investigation. A schematic illustration of these proposed antiviral mechanisms is provided in Figure 2.

### 4.2. Targeting SARS-CoV-2 Replication Machinery

Polyphenols exert antiviral activity not only by blocking viral entry but also through direct interference with the SARS-CoV-2 replication cycle. After cellular entry, SARS-CoV-2 undergoes uncoating to release its nucleocapsid and genomic RNA [45,46]. The translation of ORF1a/b generates two large polyproteins (pp1a and pp1ab) that are cleaved by two essential viral proteases—3-chymotrypsin-like protease (3CLpro/Mpro) and papain-like protease (PLpro)—to yield 16 non-structural proteins required for replication, transcription, and assembly of the replication–transcription complex [47]. Viral components are subsequently assembled in the endoplasmic reticulum–Golgi intermediate compartment and released by exocytosis [48]. Given the indispensable role of these enzymes and protein complexes in the viral life cycle, they represent highly druggable antiviral targets [49]. An expanding body of evidence indicates that tea-derived polyphenols and other plant metabolites inhibit multiple steps of SARS-CoV-2 replication, particularly through suppression of protease activity and polymerase function.

Targeting Viral Proteases (3CLpro and PLpro)

The coordination of non-structural proteins (3CLpro, PLpro and RdRp) and structural protein (S protein) plays a pivotal role in the process of replication, transcription and host cell recognition [50]. Prior research showed that extracts of Tea (Camellia sinensis) and Haritaki (Terminalia chebula) possess inhibitory effects on 3CLpro of SARS-CoV-2. Thearubigins have been proposed as bioactive constituents based on their demonstrated ability to interact with the catalytic Cys145 residue of 3CLpro, supported by protein–ligand binding analyses and protease expression studies [51].

Inhibition of this protease effectively disrupts the SARS-CoV-2 replication cycle; therefore, polyphenol-rich extracts from green tea, black tea, and Haritaki may represent mechanistically relevant candidates that warrant further experimental and clinical validation. In addition to theaflavins, EGCG has been shown to suppress replication of human coronaviruses HCoV-OC43 and HCoV-229E by reducing 3CLpro activity and decreasing viral RNA and protein expression [13]. Tea polyphenols interact with catalytic residues of 3CLpro, functioning as potential protease inhibitors and druggable targets [52]. Among tea polyphenols, EGCG exhibits the highest binding affinity for 3CLpro [53]. likely due to the presence of the 3-galloyl group (Figure 1), which provides additional hydrogen bonding and hydrophobic interactions within the catalytic pocket. Collectively, these findings highlight the potential of polyphenols to be developed as orally active protease inhibitors for COVID-19 therapy [54].

Beyond tea-derived polyphenols, a variety of natural products (including diarylheptanoids, flavonoids, and chalcones) have shown inhibitory activity against coronavirus proteases [55,56]. For instance, rutin (Figure 1) was predicted to have the strongest binding to the 3CLpro active site, while citrus and galangal polyphenols demonstrated multi-target interference with both 3CLpro and the spike–ACE2 interface [57,58]. To focus the discussion on structural motifs and SAR trends, the detailed docking parameters and interacting residues for these diverse compounds are summarized in Supplementary Table S1. Analysis of these scaffolds (Figure 1) indicates that the arrangement of hydroxyl groups and specific glycosylation patterns are key determinants of their binding stability.

Targeting Structural Viral Proteins (Spike Protein and RBD)

The SARS-CoV-2 spike (S) protein is a class I viral fusion glycoprotein that plays a central role in receptor engagement and subsequent membrane fusion [59]. Its S1 subunit is responsible for recognizing and binding the ACE2 receptor, whereas the S2 subunit facilitates fusion between viral and host membranes [60]. Because the S protein dictates viral tropism and is the key determinant of coronavirus entry, it has become a major focus of therapeutic development. Strategies directed against the S protein span monoclonal antibodies, vaccines, siRNAs, inhibitory peptides, and various small-molecule inhibitors, including certain polyphenols [59].

The potential of flavonoids to block viral entry was first noted during the SARS-CoV outbreak, where luteolin and quercetin (Figure 1) were found to interfere with spike protein engagement [61]. Subsequent screenings of various medicinal herbs (e.g., Rheum officinale) identified anthraquinones like emodin (Figure 1) as potent inhibitors of the spike–ACE2 interaction [57,62]. Interestingly, systems-pharmacology models suggest that combining these polyphenolic scaffolds with other small molecules may yield synergistic antiviral effects [63]. These findings underscore a common structural trend: the presence of specific hydroxyl patterns on flavonoid and anthraquinone cores facilitates high-affinity binding to the spike RBD, a key structural motif for developing entry inhibitors (see Supplementary Table S1 for detailed binding profiles).

Molecular docking analyses have indicated that EGCG, herbacetin, and several other flavonoids can bind with high affinity to the receptor-binding domain of the spike protein, raising the possibility that they may interfere with ACE2 engagement [64]. Additional studies have shown that hesperidin and hesperetin disrupt the spike–ACE2 interaction partly through downregulation of ACE2 and TMPRSS2 expression [65]. Naringenin has also been highlighted as a flavonoid with multiple antiviral actions, including inhibition of 3CLpro, modulation of ACE2, and anti-inflammatory activity, suggesting a potential multi-target mechanism [66]. Given its well-described antioxidant and anti-inflammatory properties, routine EGCG consumption has been proposed to help mitigate oxidative stress, cytokine storm responses, sepsis, and even pulmonary fibrosis, all of which could influence COVID-19 outcomes [67]. Other plant-derived polyphenols—such as compounds found in citrus and galangal—have likewise demonstrated *in silico* interference with RBD–ACE2 binding, pointing to possible prophylactic applications [32].

Targeting Viral RdRp

The RdRp of SARS-CoV-2 plays a central role in viral genome replication and is a well-established target for antiviral therapy. Remdesivir, an adenosine analogue, acts by being incorporated into the growing viral RNA chain, resulting in premature termination and functional inhibition of the polymerase [68,69]. Interest in polyphenol-mediated RdRp inhibition has been supported by earlier studies demonstrating antiviral activity of certain non-tea polyphenols in coronavirus models [70]. However, the clinical translation of such compounds remains limited due to poor bioavailability, prompting the development of nanoparticle-based and inhalation delivery strategies [71]. In this context, tea-derived catechins, particularly EGCG, have emerged as promising candidates due to their reported ability to interfere with viral replication machinery and their potential for improved formulation strategies.

Computational screenings have identified several additional polyphenolic scaffolds—including flavonoids and xanthones (Figure 1)—as potential inhibitors of the SARS-CoV-2 RdRp [72,73]. These *in silico* analyses indicate that the binding affinity of these compounds is largely driven by their extended aromatic systems (Figure 1) and the presence of multiple hydroxyl substitutions, which facilitate stable interaction with the RdRp catalytic site (see Supplementary Table S1 for a full list of identified compounds). Collectively, these findings support RdRp as a secondary but mechanistically relevant antiviral target for specific dietary polyphenols and natural flavonoids. A comprehensive summary of these molecular targets, along with representative polyphenols and their levels of experimental evidence, is provided in Table 1. This table outlines the principal mechanisms by which dietary polyphenols—including EGCG, luteolin, quercetin, hesperidin, theaflavins, and baicalin—modulate viral infection and host inflammatory pathways. Specifically, the synthesized evidence covers key targets such as ACE2 and TMPRSS2 for viral entry, 3CLpro and PLpro for polyprotein processing, and RdRp for genome replication, supported by data spanning in silico docking, in vitro biochemical assays, and in vivo systems. Furthermore, Table 1 incorporates the modulation of IL-6, TNF-α, NLRP3 inflammasome signaling, and oxidative stress pathways, including emerging preclinical and clinical observations from polyphenol-rich formulations and adjunctive combinations.

### 4.3. Modulation of Host Immune and Inflammatory Responses

**Figure 3:**
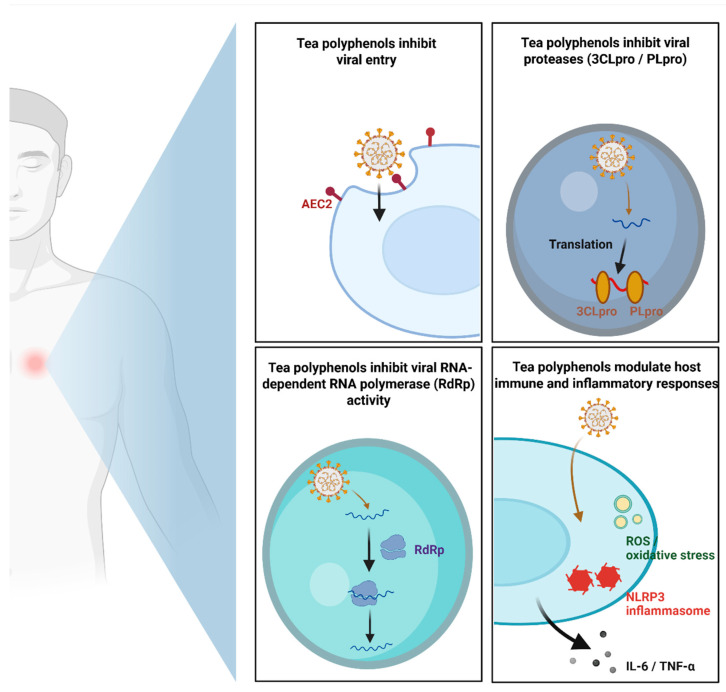
Mechanistic pathways of tea polyphenols against SARS-CoV-2 infection and inflammation. Panel illustrations summarize inhibition of viral entry (ACE2), proteases (3CLpro/PLpro), and polymerase (RdRp), alongside modulation of host inflammatory responses. Created with BioRender.com (Created in BioRender. Harrison Chang and Chi-Sheng Wu (2025) https://www.biorender.com/ accessed on 5 December 2025).

The multi-target efficacy of tea polyphenols against SARS-CoV-2 encompasses both direct antiviral interference and the modulation of host cellular environments. As synthesized in Figure 3, these compounds are proposed to reduce viral entry by lowering the binding affinity between the SARS-CoV-2 spike protein and the host ACE2 receptors on airway epithelial cells. Intracellularly, certain polyphenols interfere with the catalytic function of essential viral proteases, 3CLpro and PLpro, thereby disrupting polyprotein processing and the maturation of non-structural proteins. Furthermore, tea-derived catechins may restrict genome synthesis by inhibiting the RdRp complex. Beyond direct antiviral action, tea polyphenols significantly temper host-driven pathological processes by modulating inflammatory and oxidative pathways. This includes scavenging ROS, inhibiting NLRP3 inflammasome activation, and decreasing the systemic release of pro-inflammatory cytokines such as IL-6 and TNF-α, collectively contributing to the mitigation of severe disease progression. Severe SARS-CoV-2 infection is characterized by a dysregulated host immune response, commonly manifested as a cytokine storm that contributes to acute lung injury, respiratory failure, and multi-organ damage [74]. This hyperinflammatory state is associated with elevated levels of key mediators, including IL-6, TNF, CXCL8 (IL-8), and MCP-1, which collectively drive pathological inflammation [75]. Among these, IL-6 is frequently cited as one of the central contributors to the pathological processes associated with cytokine storms [74]. At the molecular level, this dysregulated inflammatory response is closely associated with activation of key signaling pathways, particularly NF-κB signaling and the NLRP3 inflammasome, which drive the transcription and maturation of pro-inflammatory cytokines [70].

Although several IL-6–directed biologic agents, including sarilumab, siltuximab, and tocilizumab, have been studied in clinical settings, current NIH recommendations state that the available evidence remains insufficient to support or oppose their routine use in treating COVID-19 [76]. This uncertainty has heightened interest in therapies that can temper excessive inflammation without causing broad immunosuppression. Polyphenols—well-established natural immunomodulators—have demonstrated broad anti-inflammatory effects relevant to COVID-19 pathology [77,78]. A variety of polyphenols—including curcumin, resveratrol, EGCG, emodin, naringenin, apigenin, and kaempferol—have been described in earlier work as reducing pro-inflammatory cytokine output in both cell-based and animal studies [79]. In addition, a structure–activity analysis indicated that several flavonoid scaffolds could influence NLRP3 inflammasome signaling, which is particularly relevant in settings of excessive inflammation [80]. Previous studies have demonstrated that xanthohumol exerts anti-inflammatory effects in metabolic disease models, including suppression of circulating IL-6 levels and improvement of inflammatory profiles [81].

In parallel, polyphenols can activate the Nrf2/ARE pathway through disruption of the Keap1–Nrf2 interaction, resulting in upregulation of cytoprotective genes such as *HMOX1* and *NQO1*, which mitigate oxidative stress and inflammatory injury [82]. These combined effects illustrate the pleiotropic nature of tea polyphenols, enabling simultaneous modulation of inflammatory signaling, oxidative stress responses, and immune regulation. Such multi-target actions may contribute to controlling excessive host responses and promoting recovery in COVID-19 [77].

### 4.4. Polyphenols in Tissue Repair and Post-Viral Recovery Enhancement

Beyond their antiviral activity, tea polyphenols may contribute to recovery following SARS-CoV-2 infection by modulating host tissue repair processes and promoting the resolution of inflammation [77]. Severe COVID-19 is frequently associated with acute lung injury (ALI), endothelial dysfunction, and subsequent fibrotic remodeling, all of which contribute to long-term functional impairment.

Emerging evidence indicates that polyphenols can attenuate pulmonary fibrosis through regulation of profibrotic signaling pathways. In particular, compounds such as EGCG have been shown to suppress transforming growth factor-β1 (TGFB1) signaling and reduce the expression of extracellular matrix genes, including COL1A1, thereby limiting fibroblast activation and collagen deposition [83]. In addition, polyphenols exhibit endothelial-protective effects by improving barrier integrity and reducing vascular inflammation, which are critical for restoring tissue homeostasis following viral injury [84,85].

At the mechanistic level, these recovery-enhancing effects are closely linked to the modulation of oxidative stress and immune resolution pathways, including activation of Nrf2 signaling and suppression of persistent NF-κB–mediated inflammation [82]. Through these coordinated actions, polyphenols may facilitate the transition from acute inflammation to tissue repair and remodeling.

Importantly, these mechanisms are highly relevant to post-acute sequelae of SARS-CoV-2 infection (PASC), commonly referred to as Long COVID. Persistent inflammation, endothelial dysfunction, and fibrotic changes have been implicated in prolonged respiratory, cardiovascular, and neurological symptoms. Recent studies suggest that the sustained anti-inflammatory, antioxidant, and endothelial-stabilizing properties of polyphenols may contribute to improved recovery outcomes and functional restoration in post-viral conditions [86,87].

### 4.5. Translational Perspective and Evidence Integration

Despite extensive mechanistic and *in vitro* evidence, the translation of polyphenols into clinically relevant antiviral strategies remains limited. Although extensive computational and cell-based studies point toward antiviral properties for a range of polyphenols, their behavior in SARS-CoV-2 infection models *in vivo* is still only partly understood. Several groups have proposed that polyphenols may influence the course of disease through effects on ACE2 expression [88], but relatively few animal experiments have been carried out to test this idea directly. Current evidence spans multiple levels, including *in silico* predictions, *in vitro* assays, limited *in vivo* studies, and preliminary clinical observations. One of the more informative studies examined Pudilan Xiaoyan Oral Liquid (PDL)—a traditional Chinese formulation containing more than 180 phytochemicals—and reported reductions in viral burden together with improvements in clinical features in hACE2 transgenic mice infected with SARS-CoV-2 [89]. These findings offer preliminary support for the notion that multi-component botanical preparations might provide therapeutic benefit.

In addition to preclinical evidence, early clinical observations highlight the translational potential of polyphenol-based interventions. A report described substantial clinical improvement in a patient with SARS-CoV-2–induced multifocal pneumonia following administration of a nebulized formulation containing quercetin and N-acetylcysteine, adjunctive to standard therapy with hydroxychloroquine and antibiotics [90]. Although anecdotal, this case emphasizes the importance of further therapeutic evaluation of polyphenol-based nutraceuticals in controlled clinical trials.

High-throughput and computational screening continue to accelerate the discovery of polyphenolic antiviral leads. However, a major limitation lies in the restricted availability of curated polyphenol compound libraries. Screening of polyphenol-rich plant extracts remains widely used but presents challenges, including difficulty identifying active constituents and potential antagonistic interactions among components.

Recent improvements in bioassay-guided fractionation, together with growing use of machine-learning methods for dereplication, have begun to ease some of these obstacles. These tools allow researchers to identify active constituents earlier in the discovery pipeline, in some cases before full chemical purification is complete. Their utility is further enhanced by the expansion of metabolomics resources and curated reference databases, including Phenol-Explorer [91], KNApSAcK [92], and the Global Natural Products Social (GNPS) molecular networking platform [93], which facilitate compound annotation and dereplication in complex polyphenol mixtures.

Considering these developments as a whole, polyphenols appear to represent a promising but still underexplored class of antiviral candidates. Future progress is likely to depend on integrating experimental virology with computational methodologies, including bioinformatics, metabolomics, and systems-pharmacology approaches. A more unified framework could clarify how these naturally occurring compounds might be advanced toward realistic therapeutic or prophylactic applications for SARS-CoV-2 and other viruses with pandemic potential. Collectively, these findings highlight the need to bridge mechanistic insights with clinically actionable strategies, emphasizing the importance of integrated translational frameworks.

**Table 1:** Molecular targets and mechanisms of tea polyphenols against SARS-CoV-2.

Mechanism	Molecular Target(s)	Representative Polyphenols	Evidence	Key References
Inhibition of Viral Entry	ACE2, Spike RBD, TMPRSS2	EGCG, Eriodictyol, Luteolin, Quercetin, Hesperidin, Resveratrol	*In vitro*, *In silico*	[35,36,37,38,77,78,79,80,81,82,,83,84]
Protease Inhibition	3CLpro (Mpro), PLpro	EGCG, Theaflavins, Rutin, Chalcones, Kaempferol	*In vitro*, *In silico*	[62,63,64,65,66,67,68,69,70,71,72,73]
Inhibition of RdRp	RNA-dependent RNA polymerase	EGCG, Resveratrol, Baicalin, Myricetin, Quercetagetin	*In vitro*, *In silico*	[45,85,86,87,88,89,90,91]
Modulation of Immune Response	IL-6, TNF-α, NLRP3 inflammasome, oxidative stress pathways	Curcumin, Resveratrol, EGCG, Naringenin, Apigenin	*In vitro*, *In vivo*	[92,93,94,95,96,97,98,99]
Systemic/Preclinical/Clinical Evidence	Multi-target	PDL (TCM), Quercetin + NAC	Animal models, Case reports	[100,101,102,103,104,105]

Note: This table summarizes the pleiotropic effects of representative tea polyphenols on viral entry, replication, and host inflammatory signaling based on in silico, in vitro, and emerging clinical evidence.

### 4.6. Pleiotropic Effects: Opportunities and Translational Challenges

The pleiotropic nature of tea polyphenols, characterized by their ability to modulate multiple viral and host pathways simultaneously, is often considered a key advantage. These compounds have been reported to interact with diverse molecular targets, including viral proteases, host cell receptors, and inflammatory signaling pathways [77].

However, this multi-target activity also introduces significant translational complexity. First, it complicates clinical trial design, as it becomes challenging to define specific endpoints that capture the full spectrum of biological effects. Second, dose selection becomes less straightforward, since different pathways may require distinct concentration ranges for modulation. Third, attributing clinical observational outcomes to specific mechanisms of action is inherently difficult in the context of pleiotropic compounds.

Rather than representing a limitation, these challenges highlight the need for more integrative research approaches. Future studies should adopt system-level strategies, incorporating multi-omics analyses and biomarker-driven trial designs to better capture the complex biological effects of polyphenols. Such approaches may provide a more accurate assessment of their therapeutic potential in complex diseases such as COVID-19 [94].

## 5. Bridging the Preclinical-to-Clinical Gap: Stratifying the Evidence Hierarchy

**Figure 4:**
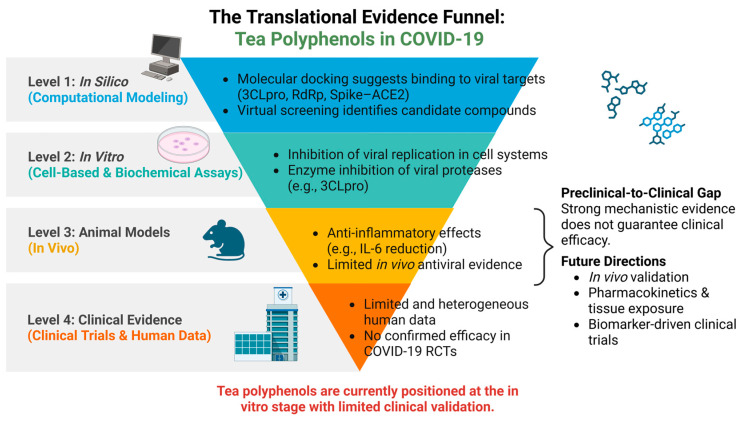
The translational evidence hierarchy funnel for tea polyphenols. The funnel visualizes the diminishing volume of evidence as research moves from computational screening to clinical validation, highlighting the translational bottleneck. Detailed critical analysis of this hierarchy is provided in Section 5. Created with BioRender.com (accessed on 22 March 2026).

Although tea polyphenols have demonstrated a wide range of antiviral and immunomodulatory activities, it is imperative to critically evaluate the strength and translational relevance of the available evidence. As conceptualized in the translational evidence hierarchy funnel (Figure 4), current findings are predominantly concentrated at the upper levels, derived from *in silico* molecular docking studies and in vitro enzymatic or cell-based assays. While these are valuable for mechanistic exploration, they do not necessarily predict clinical efficacy. At these foundational levels, tea polyphenols provide abundant mechanistic evidence, including predicted binding to viral targets and inhibition of viral replication. However, moving down the funnel toward the in vivo domain, studies remain limited and primarily demonstrate anti-inflammatory effects with less consistent antiviral validation.

The COVID-19 pandemic has provided multiple precedents—such as hydroxychloroquine, high-dose vitamin C, and certain flavonoids—where exceptionally strong preclinical signals failed to translate into meaningful clinical benefit in randomized controlled trials (RCTs) [95,96]. This distribution underscores a substantial translational gap between mechanistic promise and clinical applicability. At the clinical apex of the funnel, available human data are currently limited and heterogeneous, with no confirmed efficacy in large-scale RCTs. This hierarchical framework emphasizes that strong mechanistic evidence does not inherently equate to clinical success. Bridging this gap will require systematic validation across progressively more complex biological systems. Consequently, future research should transition from simple molecular screening toward more integrative strategies, including pharmacokinetic and tissue distribution studies, biomarker-guided clinical trial designs, and multi-target evaluation frameworks that better capture the pleiotropic nature of tea polyphenols.

## 6. Bioavailability and Pharmacokinetic Limitations

Arguably the most formidable translational barrier for tea polyphenols is their inherently poor systemic bioavailability [97]. While *in vitro* studies consistently demonstrate robust viral inhibition and immunomodulation, these effects often require concentrations that are pharmacokinetically unattainable *in vivo* via standard oral consumption. Following oral administration, the peak plasma concentration of catechins like EGCG typically ranges only between 0.1 and 1 µM. This is frequently an order of magnitude lower than the half-maximal inhibitory concentrations (IC50) required to effectively neutralize SARS-CoV-2 targets such as 3CLpro or RdRp in cell-based assays [98].

This discrepancy stems from extensive pharmacokinetic hurdles. Upon ingestion, EGCG undergoes rapid Phase II hepatic metabolism, including methylation, glucuronidation, and sulfation [99]. Furthermore, polyphenols are highly susceptible to auto-oxidation in alkaline physiological fluids and face significant efflux transport via multidrug resistance-associated proteins (MRPs) [100]. Consequently, these factors severely limit systemic exposure and result in highly uncertain tissue penetration, particularly into the lung epithelial lining fluid, the primary site of SARS-CoV-2 replication and respiratory injury. Without addressing these metabolic barriers, purely mechanistic claims risk biological irrelevance at actual human dosing levels [101].

To bridge this critical gap, contemporary research must pivot from discovering new *in vitro* targets to developing advanced delivery platforms. Recent pharmaceutical innovations have proposed nanoformulations—such as liposomal encapsulation, lipid nanoparticles (LNPs), and polymeric nanocarriers—to protect catechins from premature degradation and enhance cellular uptake [102]. Moreover, aerosolized and inhaled delivery systems represent a highly promising translational solution. By administering polyphenols directly to the respiratory tract via nebulizers or dry powder inhalers, these localized delivery methods can entirely bypass hepatic first-pass metabolism, directly achieving therapeutically relevant concentrations in the pulmonary mucosa while minimizing systemic off-target effects [103].

## 7. Nutraceutical Potential of Tea Polyphenols

Tea products, including green, oolong, and black tea, are widely consumed worldwide and are increasingly formulated into functional beverages [104,105]. As daily dietary supplements, they are attractive because they are inexpensive, culturally acceptable, and can be integrated into habitual drinking patterns while targeting cardiometabolic risk, immune function, and respiratory infections. Clinical and pharmacokinetic data indicate that a typical 200–250 mL cup of green tea provides roughly 50–100 mg EGCG, with estimated habitual intakes of 90–300 mg EGCG/day in regular tea drinkers [106].

From a safety perspective, multiple regulatory and systematic reviews converge on the liver as the critical target organ for potential adverse effects of concentrated green tea extracts, particularly when administered as high-dose bolus capsules under fasting conditions. A systematic review by Hu and colleagues evaluated the safety profile of green tea preparations and found that both brewed tea and beverage-type extracts are generally well tolerated. Drawing on pooled data, the authors suggested that an intake of about 338 mg of EGCG per day is acceptable when delivered in solid supplement form, while higher amounts—up to roughly 704 mg per day—appear safe when consumed as part of a tea beverage [107]. More recent evidence and regulatory assessments have largely reinforced these findings while providing additional nuance. Subsequent studies indicate that hepatotoxicity associated with EGCG remains rare but is more likely to occur at high doses, particularly when consumed as concentrated supplements rather than as traditional tea infusions [108]. In addition, recent evaluations emphasize that dosing pattern and formulation significantly influence safety, with bolus intake posing a higher risk compared to distributed intake through beverages [109]. These updates highlight the continued relevance of EGCG safety considerations and underscore the importance of dose optimization in translational and clinical applications. These observations are consistent with current regulatory perspectives emphasizing the importance of dose, formulation, and exposure conditions in determining EGCG safety [110]. These conclusions are consistent with those of the European Food Safety Authority, which has reported that liver-related adverse events have been associated mainly with high-dose, concentrated supplements rather than with customary brewed tea [111]. A systematic review of randomized controlled trials found no consistent liver enzyme elevation at moderate catechin doses typical of nutraceutical use, further supporting an acceptable safety margin when dosing is kept within these ranges. Pharmacokinetic trials of purified EGCG and decaffeinated green tea extract (Polyphenon E) in healthy adults suggest that repeated oral doses of 400–800 mg/day EGCG are generally well tolerated, with mainly mild gastrointestinal side effects.

As nutraceuticals, tea polyphenols have also been evaluated for their ability to prevent viral respiratory infections, which is directly relevant to COVID-19 risk modulation. A randomized, placebo-controlled trial in healthcare workers showed that 12-week consumption of a catechin-containing beverage (three daily doses, total 171 mg catechins/day) was associated with a lower incidence of acute upper respiratory infections compared with placebo (hazard ratio 0.46 vs. placebo) [112]. A recent systematic review and meta-analysis pooling six randomized trials and four cohort studies concluded that tea and tea catechin consumption (as drinks or capsules) significantly reduced the risk of influenza and acute upper respiratory tract infections (overall risk ratio 0.74, 95% CI 0.64–0.87), with evidence of a dose–response relationship between daily catechin intake and infection risk reduction [113]. These data, although not COVID-specific, support a potential association between tea polyphenol intake and reduced risk of respiratory infections, although causality and applicability to COVID-19 remain uncertain.

Tea polyphenols may have additional value when they are taken together with certain micronutrients that already appear in many immune-support formulas, such as vitamins C and D or zinc. Vitamins C and D influence several branches of immune response, help manage oxidative stress, and play a role in maintaining epithelial health, while zinc is involved in antiviral defenses, ACE2 regulation, and interferon signaling [114,115]. There has also been some discussion about whether EGCG might work as a zinc ionophore, which could raise intracellular zinc levels and potentially reinforce zinc-dependent antiviral pathways. Clinical data remain limited, but one small case series in people dealing with post-COVID symptoms evaluated a supplement that combined green-tea extract (75 mg per capsule), vitamin C, vitamin D_3_, and zinc, taken as three capsules twice daily for four months. The participants showed decreases in d-dimer, IL-6, and NT-proBNP, along with gradual improvements in several quality-of-life measures, and no major safety problems were reported [116]. Even with its small sample size and lack of a control group, the study suggests how polyphenols might fit into broader multi-nutrient approaches intended to support immune function and help ease cardiometabolic stress after viral illness.

Another point that should be considered is the proper formulation for these polyphenol-based nutraceuticals. To counteract low bioavailability concerns and increase concentrations of active polyphenols in the respiratory tract, the primary site of infection, strategies such as nano-formulations, targeted delivery platforms, and aerosol-based administration (e.g., nebulizers, inhalers) have been proposed as promising approaches [117].

Overall, the available evidence indicates that tea polyphenols are generally well tolerated and easy to include in everyday dietary habits, whether through regular tea drinking or moderate extract use. Their potential to lower the risk of respiratory infections and to reduce systemic inflammation may also be strengthened when they are taken alongside micronutrients like vitamins C and D or zinc. That said, when intake goes beyond what people normally consume in their diets and moves into the higher amounts found in supplements, it becomes important to think more carefully about the appropriate dose, the quality of the formulation, and any possible effects on the liver.

## 8. Future Research Priorities

To address the aforementioned translational gaps and pharmacokinetic limitations, future research should adopt a more integrated and clinically oriented framework to advance tea polyphenols toward therapeutic application. First, validation studies using live SARS-CoV-2 and emerging variants remain essential. Although multiple studies have shown that EGCG and related tea polyphenols can inhibit SARS-CoV-2 infection, including variant-associated spike-mediated entry, much of the available evidence is still based on *in vitro*, pseudovirus, or limited authentic-virus systems. Therefore, broader confirmation using authentic SARS-CoV-2 strains and currently circulating variants is needed to establish robustness under physiologically relevant conditions [118,119].

Second, pharmacokinetic and pharmacodynamic studies should be prioritized, particularly those addressing target-site exposure in the respiratory tract. Poor systemic bioavailability remains a major obstacle for polyphenols, and current evidence supports the broader need for improved delivery strategies, including nano-delivery systems and local pulmonary administration, to enhance stability, tissue distribution, and biological efficacy [120]. Because SARS-CoV-2 primarily affects the airway and lung epithelium, future studies should quantify concentrations in pulmonary tissue and, where feasible, epithelial lining fluid, rather than relying solely on plasma pharmacokinetics [121].

Third, early-phase clinical studies should move beyond conventional single-endpoint designs and incorporate biomarker-driven outcome frameworks. Consensus efforts for COVID-19 trials have emphasized viral burden, survival, and clinical progression as core outcomes, while more recent biomarker studies suggest that inflammatory mediators such as IL-6, CRP, and TNF-α may be useful adjunctive endpoints for capturing host-response modulation, especially in interventions with pleiotropic mechanisms [122]. Accordingly, Phase I/II studies of tea polyphenols should integrate virological, clinical, and inflammatory endpoints to better define mechanism-response relationships and support patient stratification [123].

Finally, long COVID represents an important yet underexplored application area. Persistent inflammation, immune dysregulation, and fibrotic remodeling are increasingly recognized as important components of post-COVID sequelae, and biomarker studies have repeatedly implicated inflammatory pathways, including IL-6, CRP, TNF-α, and TGF-β, in ongoing symptomatology [124]. In parallel, mechanistic reviews continue to support a role for NLRP3 inflammasome activation in COVID-19 pathogenesis and its longer-term inflammatory consequences, making this pathway a rational target for polyphenol-based interventions [125]. Thus, future studies should evaluate whether tea polyphenols can modulate chronic inflammatory and profibrotic pathways relevant to long COVID, particularly in pulmonary and systemic post-viral syndromes.

Collectively, these priorities provide a practical translational roadmap linking mechanistic evidence with future preclinical and clinical development. By integrating authentic-virus validation, respiratory PK/PD profiling, biomarker-guided trial design, and long COVID-oriented applications, future work may better define the therapeutic potential of tea polyphenols in COVID-19 and related post-viral conditions.

## 9. Conclusions

Although COVID-19 is no longer considered an emerging infectious disease, it continues to impose a significant burden on vulnerable populations. In this context, tea polyphenols have emerged as potential adjunctive candidates that may complement existing antiviral and immunomodulatory therapies. The foregoing review of the literature demonstrates that tea polyphenols have increasingly gained attention but are still not fully characterized through systematic preclinical and clinical investigations for potential antiviral effects against SARS-CoV-2. Nonetheless, their clinical translation remains limited by key challenges, including poor bioavailability, insufficient target-site exposure, and the complexity of multi-target mechanisms. At the molecular level, tea polyphenols demonstrate the ability to interact with viral proteases and host targets; however, these findings are primarily derived from preclinical studies.

While tea polyphenols demonstrate promising antiviral and immunomodulatory mechanisms, current evidence remains insufficient to support clinical efficacy in COVID-19. No large-scale randomized controlled trials have demonstrated therapeutic benefit to date. Therefore, tea polyphenols should be positioned as mechanistically relevant adjunctive modulators rather than established therapeutic agents, highlighting the need for integrative, biomarker-driven, and clinically oriented research strategies to bridge the current translational gap. Ultimately, their therapeutic potential may depend less on direct antiviral potency and more on their ability to modulate the host–virus interface in a multi-target and context-dependent manner.

## Data Availability

No new data were created or analyzed in this study. Data sharing is not applicable to this article.

## Supplementary Materials

The following supporting information can be downloaded at: https://www.mdpi.com/article/10.3390/cimb48040379/s1.

## References

[1] van Doremalen N., Bushmaker T., Morris D.H., Holbrook M.G., Gamble A., Williamson B.N., Tamin A., Harcourt J.L., Thornburg N.J., Gerber S.I. (2020). Aerosol and Surface Stability of SARS-CoV-2 as Compared with SARS-CoV-1. N. Engl. J. Med..

[2] Sorensen M.D., Sorensen B., Gonzalez-Dosal R., Melchjorsen C.J., Weibel J., Wang J., Chen W.J., Yang H., Kristensen P. (2006). Severe acute respiratory syndrome (SARS): Development of diagnostics and antivirals. Ann. N. Y. Acad. Sci..

[3] Hu B., Guo H., Zhou P., Shi Z.L. (2021). Characteristics of SARS-CoV-2 and COVID-19. Nat. Rev. Microbiol..

[4] Zhang A.R., Shi W.Q., Liu K., Li X.L., Liu M.J., Zhang W.H., Zhao G.P., Chen J.J., Zhang X.A., Miao D. (2021). Epidemiology and evolution of Middle East respiratory syndrome coronavirus, 2012–2020. Infect. Dis. Poverty.

[5] Deng S.Q., Peng H.J. (2020). Characteristics of and Public Health Responses to the Coronavirus Disease 2019 Outbreak in China. J. Clin. Med..

[6] Gandhi R.T., Lynch J.B., Del Rio C. (2020). Mild or Moderate COVID-19. N. Engl. J. Med..

[7] Williamson E.J., Walker A.J., Bhaskaran K., Bacon S., Bates C., Morton C.E., Curtis H.J., Mehrkar A., Evans D., Inglesby P. (2020). Factors associated with COVID-19-related death using OpenSAFELY. Nature.

[8] Lian J., Jin X., Hao S., Cai H., Zhang S., Zheng L., Jia H., Hu J., Gao J., Zhang Y. (2020). Analysis of Epidemiological and Clinical Features in Older Patients With Coronavirus Disease 2019 (COVID-19) Outside Wuhan. Clin. Infect. Dis..

[9] Planas D., Veyer D., Baidaliuk A., Staropoli I., Guivel-Benhassine F., Rajah M.M., Planchais C., Porrot F., Robillard N., Puech J. (2021). Reduced sensitivity of SARS-CoV-2 variant Delta to antibody neutralization. Nature.

[10] Nalbandian A., Sehgal K., Gupta A., Madhavan M.V., McGroder C., Stevens J.S., Cook J.R., Nordvig A.S., Shalev D., Sehrawat T.S. (2021). Post-acute COVID-19 syndrome. Nat. Med..

[11] Davis H.E., McCorkell L., Vogel J.M., Topol E.J. (2023). Long COVID: Major findings, mechanisms and recommendations. Nat. Rev. Microbiol..

[12] Ohgitani E., Shin-Ya M., Ichitani M., Kobayashi M., Takihara T., Kawamoto M., Kinugasa H., Mazda O. (2021). Significant Inactivation of SARS-CoV-2 In Vitro by a Green Tea Catechin, a Catechin-Derivative, and Black Tea Galloylated Theaflavins. Molecules.

[13] Jang M., Park R., Park Y.I., Cha Y.E., Yamamoto A., Lee J.I., Park J. (2021). EGCG, a green tea polyphenol, inhibits human coronavirus replication in vitro. Biochem. Biophys. Res. Commun..

[14] Cory H., Passarelli S., Szeto J., Tamez M., Mattei J. (2018). The Role of Polyphenols in Human Health and Food Systems: A Mini-Review. Front. Nutr..

[15] Cecchini R., Cecchini A.L. (2020). SARS-CoV-2 infection pathogenesis is related to oxidative stress as a response to aggression. Med. Hypotheses.

[16] Merad M., Martin J.C. (2020). Pathological inflammation in patients with COVID-19: A key role for monocytes and macrophages. Nat. Rev. Immunol..

[17] Shaukat H., Ali A., Zhang Y., Ahmad A., Riaz S., Khan A., Mehany T., Qin H. (2023). Tea polyphenols: Extraction techniques and its potency as a nutraceutical. Front. Sustain. Food Syst..

[18] Huang H., Fan B., Wei C., Song Y., Jiang W., Huang Q., Deng D., Wang F., Yao M. (2026). Polyphenols as Modulators of Macrophage Polarization: Mechanisms and Therapeutic Potential in Chronic Inflammatory Diseases. Phytother. Res..

[19] LeBlanc E.V., Colpitts C.C. (2022). The green tea catechin EGCG provides proof-of-concept for a pan-coronavirus attachment inhibitor. Sci. Rep..

[20] Kaul R., Paul P., Kumar S., Busselberg D., Dwivedi V.D., Chaari A. (2021). Promising Antiviral Activities of Natural Flavonoids against SARS-CoV-2 Targets: Systematic Review. Int. J. Mol. Sci..

[21] Yang C.C., Wu C.J., Chien C.Y., Chien C.T. (2021). Green Tea Polyphenol Catechins Inhibit Coronavirus Replication and Potentiate the Adaptive Immunity and Autophagy-Dependent Protective Mechanism to Improve Acute Lung Injury in Mice. Antioxidants.

[22] Mokra D., Adamcakova J., Mokry J. (2022). Green Tea Polyphenol (-)-Epigallocatechin-3-Gallate (EGCG): A Time for a New Player in the Treatment of Respiratory Diseases?. Antioxidants.

[23] Ohgitani E., Shin-Ya M., Ichitani M., Kobayashi M., Takihara T., Kawamoto M., Kinugasa H., Mazda O. (2021). Rapid Inactivation In Vitro of SARS-CoV-2 in Saliva by Black Tea and Green Tea. Pathogens.

[24] Du A., Zheng R., Disoma C., Li S., Chen Z., Li S., Liu P., Zhou Y., Shen Y., Liu S. (2021). Epigallocatechin-3-gallate, an active ingredient of Traditional Chinese Medicines, inhibits the 3CLpro activity of SARS-CoV-2. Int. J. Biol. Macromol..

[25] Bahun M., Jukic M., Oblak D., Kranjc L., Bajc G., Butala M., Bozovicar K., Bratkovic T., Podlipnik C., Poklar Ulrih N. (2022). Inhibition of the SARS-CoV-2 3CL^pro^ main protease by plant polyphenols. Food Chem..

[26] Letko M., Marzi A., Munster V. (2020). Functional assessment of cell entry and receptor usage for SARS-CoV-2 and other lineage B betacoronaviruses. Nat. Microbiol..

[27] Peacock T.P., Goldhill D.H., Zhou J., Baillon L., Frise R., Swann O.C., Kugathasan R., Penn R., Brown J.C., Sanchez-David R.Y. (2021). The furin cleavage site in the SARS-CoV-2 spike protein is required for transmission in ferrets. Nat. Microbiol..

[28] Hoffmann M., Kleine-Weber H., Schroeder S., Kruger N., Herrler T., Erichsen S., Schiergens T.S., Herrler G., Wu N.H., Nitsche A. (2020). SARS-CoV-2 Cell Entry Depends on ACE2 and TMPRSS2 and Is Blocked by a Clinically Proven Protease Inhibitor. Cell.

[29] Poduri R., Joshi G., Jagadeesh G. (2020). Drugs targeting various stages of the SARS-CoV-2 life cycle: Exploring promising drugs for the treatment of COVID-19. Cell. Signal..

[30] Abubakar M.B., Usman D., El-Saber Batiha G., Cruz-Martins N., Malami I., Ibrahim K.G., Abubakar B., Bello M.B., Muhammad A., Gan S.H. (2021). Natural Products Modulating Angiotensin Converting Enzyme 2 (ACE2) as Potential COVID-19 Therapies. Front. Pharmacol..

[31] Hong M., Cheng L., Liu Y., Wu Z., Zhang P., Zhang X. (2022). A Natural Plant Source-Tea Polyphenols, a Potential Drug for Improving Immunity and Combating Virus. Nutrients.

[32] Bizzoca M.E., Leuci S., Mignogna M.D., Muzio E.L., Caponio V.C.A., Muzio L.L. (2022). Natural Compounds May Contribute in Preventing SARS-CoV-2 Infection: A Narrative Review. Food Sci. Hum. Wellness.

[33] Xu L., Ho C.T., Liu Y., Wu Z., Zhang X. (2022). Potential Application of Tea Polyphenols to the Prevention of COVID-19 Infection: Based on the Gut-Lung Axis. Front. Nutr..

[34] Sayed A.M., Khattab A.R., AboulMagd A.M., Hassan H.M., Rateb M.E., Zaid H., Abdelmohsen U.R. (2020). Nature as a treasure trove of potential anti-SARS-CoV drug leads: A structural/mechanistic rationale. RSC Adv..

[35] Horne J.R., Vohl M.C. (2020). Biological plausibility for interactions between dietary fat, resveratrol, ACE2, and SARS-CoV illness severity. Am. J. Physiol. Endocrinol. Metab..

[36] Cheng F.J., Ho C.Y., Li T.S., Chen Y., Yeh Y.L., Wei Y.L., Huynh T.K., Chen B.R., Ko H.Y., Hsueh C.S. (2023). Umbelliferone and eriodictyol suppress the cellular entry of SARS-CoV-2. Cell Biosci..

[37] Shanmugarajan D., P P., Kumar B.R.P., Suresh B. (2020). Curcumin to inhibit binding of spike glycoprotein to ACE2 receptors: Computational modelling, simulations, and ADMET studies to explore curcuminoids against novel SARS-CoV-2 targets. RSC Adv..

[38] Jena A.B., Kanungo N., Nayak V., Chainy G.B.N., Dandapat J. (2021). Catechin and curcumin interact with S protein of SARS-CoV2 and ACE2 of human cell membrane: Insights from computational studies. Sci. Rep..

[39] Yamaguchi T., Hoshizaki M., Minato T., Nirasawa S., Asaka M.N., Niiyama M., Imai M., Uda A., Chan J.F.-W., Takahashi S. (2021). ACE2-like carboxypeptidase B38-CAP protects from SARS-CoV-2-induced lung injury. Nat. Commun..

[40] Batlle D., Wysocki J., Satchell K. (2020). Soluble angiotensin-converting enzyme 2: A potential approach for coronavirus infection therapy?. Clin. Sci..

[41] Zoufaly A., Poglitsch M., Aberle J.H., Hoepler W., Seitz T., Traugott M., Grieb A., Pawelka E., Laferl H., Wenisch C. (2020). Human recombinant soluble ACE2 in severe COVID-19. Lancet Respir. Med..

[42] Kim H.R., Kim W.K., Ha A.W. (2019). Effects of Phytochemicals on Blood Pressure and Neuroprotection Mediated Via Brain Renin-Angiotensin System. Nutrients.

[43] Rattis B.A.C., Ramos S.G., Celes M.R.N. (2021). Curcumin as a Potential Treatment for COVID-19. Front. Pharmacol..

[44] Wu X., Yang M., Zhang H., Yang L., He Y., Cheng X., Zhu G. (2025). Advances in Understanding Renin-Angiotensin System-Mediated Anti-Tumor Activity of Natural Polyphenols. Biomolecules.

[45] Yang M., Wei J., Huang T., Lei L., Shen C., Lai J., Yang M., Liu L., Yang Y., Liu G. (2021). Resveratrol inhibits the replication of severe acute respiratory syndrome coronavirus 2 (SARS-CoV-2) in cultured Vero cells. Phytother. Res..

[46] Zhang Z., Zhang X., Bi K., He Y., Yan W., Yang C.S., Zhang J. (2021). Potential protective mechanisms of green tea polyphenol EGCG against COVID-19. Trends Food Sci. Technol..

[47] V’Kovski P., Gerber M., Kelly J., Pfaender S., Ebert N., Braga Lagache S., Simillion C., Portmann J., Stalder H., Gaschen V. (2019). Determination of host proteins composing the microenvironment of coronavirus replicase complexes by proximity-labeling. eLife.

[48] V’Kovski P., Kratzel A., Steiner S., Stalder H., Thiel V. (2021). Coronavirus biology and replication: Implications for SARS-CoV-2. Nat. Rev. Microbiol..

[49] Dai W., Zhang B., Jiang X.M., Su H., Li J., Zhao Y., Xie X., Jin Z., Peng J., Liu F. (2020). Structure-based design of antiviral drug candidates targeting the SARS-CoV-2 main protease. Science.

[50] Murugan N.A., Pandian C.J., Jeyakanthan J. (2021). Computational investigation on Andrographis paniculata phytochemicals to evaluate their potency against SARS-CoV-2 in comparison to known antiviral compounds in drug trials. J. Biomol. Struct. Dyn..

[51] Upadhyay S., Tripathi P.K., Singh M., Raghavendhar S., Bhardwaj M., Patel A.K. (2020). Evaluation of medicinal herbs as a potential therapeutic option against SARS-CoV-2 targeting its main protease. Phytother. Res..

[52] Mhatre S., Naik S., Patravale V. (2021). A molecular docking study of EGCG and theaflavin digallate with the druggable targets of SARS-CoV-2. Comput. Biol. Med..

[53] Ghosh R., Chakraborty A., Biswas A., Chowdhuri S. (2021). Evaluation of green tea polyphenols as novel corona virus (SARS CoV-2) main protease (Mpro) inhibitors—An in silico docking and molecular dynamics simulation study. J. Biomol. Struct. Dyn..

[54] Namchuk M.N. (2021). Early Returns on Small Molecule Therapeutics for SARS-CoV-2. ACS Infect. Dis..

[55] Ullrich S., Nitsche C. (2020). The SARS-CoV-2 main protease as drug target. Bioorg. Med. Chem. Lett..

[56] Das S., Sarmah S., Lyndem S., Singha Roy A. (2021). An investigation into the identification of potential inhibitors of SARS-CoV-2 main protease using molecular docking study. J. Biomol. Struct. Dyn..

[57] Mani J.S., Johnson J.B., Steel J.C., Broszczak D.A., Neilsen P.M., Walsh K.B., Naiker M. (2020). Natural product-derived phytochemicals as potential agents against coronaviruses: A review. Virus Res..

[58] Jo S., Kim S., Shin D.H., Kim M.S. (2020). Inhibition of SARS-CoV 3CL protease by flavonoids. J. Enzym. Inhib. Med. Chem..

[59] Walls A.C., Park Y.J., Tortorici M.A., Wall A., McGuire A.T., Veesler D. (2020). Structure, Function, and Antigenicity of the SARS-CoV-2 Spike Glycoprotein. Cell.

[60] Jackson C.B., Farzan M., Chen B., Choe H. (2022). Mechanisms of SARS-CoV-2 entry into cells. Nat. Rev. Mol. Cell Biol..

[61] Kim C.H. (2021). Anti-SARS-CoV-2 Natural Products as Potentially Therapeutic Agents. Front. Pharmacol..

[62] Ho T.Y., Wu S.L., Chen J.C., Li C.C., Hsiang C.Y. (2007). Emodin blocks the SARS coronavirus spike protein and angiotensin-converting enzyme 2 interaction. Antivir. Res..

[63] Zhou Y., Hou Y., Shen J., Huang Y., Martin W., Cheng F. (2020). Network-based drug repurposing for novel coronavirus 2019-nCoV/SARS-CoV-2. Cell Discov..

[64] Tallei T.E., Tumilaar S.G., Niode N.J., Fatimawali, Kepel B.J., Idroes R., Effendi Y., Sakib S.A., Emran T.B. (2020). Potential of Plant Bioactive Compounds as SARS-CoV-2 Main Protease (M^pro^) and Spike (S) Glycoprotein Inhibitors: A Molecular Docking Study. Scientifica.

[65] Cheng F.J., Huynh T.K., Yang C.S., Hu D.W., Shen Y.C., Tu C.Y., Wu Y.C., Tang C.H., Huang W.C., Chen Y. (2021). Hesperidin Is a Potential Inhibitor against SARS-CoV-2 Infection. Nutrients.

[66] Ubani A., Agwom F., RuthMorenikeji O., Nathan S., Luka P., Umera A., Umar U., Omale S., Nnadi N.E., Aguiyi J.C. (2020). Molecular Docking Analysis of Some Phytochemicals on Two SARS-CoV-2 Targets: Potential Lead Compounds Against Two Target Sites of SARS-CoV-2 Obtained from Plants. bioRxiv.

[67] Gil C., Ginex T., Maestro I., Nozal V., Barrado-Gil L., Cuesta-Geijo M.A., Urquiza J., Ramirez D., Alonso C., Campillo N.E. (2020). COVID-19: Drug Targets and Potential Treatments. J. Med. Chem..

[68] Hendaus M.A. (2021). Remdesivir in the treatment of coronavirus disease 2019 (COVID-19): A simplified summary. J. Biomol. Struct. Dyn..

[69] Tchesnokov E.P., Feng J.Y., Porter D.P., Gotte M. (2019). Mechanism of Inhibition of Ebola Virus RNA-Dependent RNA Polymerase by Remdesivir. Viruses.

[70] McKee D.L., Sternberg A., Stange U., Laufer S., Naujokat C. (2020). Candidate drugs against SARS-CoV-2 and COVID-19. Pharmacol. Res..

[71] Marinella M.A. (2020). Indomethacin and resveratrol as potential treatment adjuncts for SARS-CoV-2/COVID-19. Int. J. Clin. Pract..

[72] Wu C., Liu Y., Yang Y., Zhang P., Zhong W., Wang Y., Wang Q., Xu Y., Li M., Li X. (2020). Analysis of therapeutic targets for SARS-CoV-2 and discovery of potential drugs by computational methods. Acta Pharm. Sin. B.

[73] Singh S., Sk M.F., Sonawane A., Kar P., Sadhukhan S. (2021). Plant-derived natural polyphenols as potential antiviral drugs against SARS-CoV-2 via RNA-dependent RNA polymerase (RdRp) inhibition: An in-silico analysis. J. Biomol. Struct. Dyn..

[74] Omolo C.A., Soni N., Fasiku V.O., Mackraj I., Govender T. (2020). Update on therapeutic approaches and emerging therapies for SARS-CoV-2 virus. Eur. J. Pharmacol..

[75] Zumla A., Hui D.S., Azhar E.I., Memish Z.A., Maeurer M. (2020). Reducing mortality from 2019-nCoV: Host-directed therapies should be an option. Lancet.

[76] Atal S., Fatima Z. (2020). IL-6 Inhibitors in the Treatment of Serious COVID-19: A Promising Therapy?. Pharmaceut. Med..

[77] Paraiso I.L., Revel J.S., Stevens J.F. (2020). Potential use of polyphenols in the battle against COVID-19. Curr. Opin. Food Sci..

[78] Ferreira C., Vieira P., Sa H., Malva J., Castelo-Branco M., Reis F., Viana S. (2024). Polyphenols: Immunonutrients tipping the balance of immunometabolism in chronic diseases. Front. Immunol..

[79] Godos J., Carota G., Caruso G., Micek A., Frias-Toral E., Giampieri F., Brito-Ballester J., Rodriguez Velasco C.L., Quiles J.L., Battino M. (2025). Molecular mechanisms underlying the neuroprotective effects of polyphenols: Implications for cognitive function. EXCLI J..

[80] Lim H., Min D.S., Park H., Kim H.P. (2018). Flavonoids interfere with NLRP3 inflammasome activation. Toxicol. Appl. Pharmacol..

[81] Miranda C.L., Elias V.D., Hay J.J., Choi J., Reed R.L., Stevens J.F. (2016). Xanthohumol improves dysfunctional glucose and lipid metabolism in diet-induced obese C57BL/6J mice. Arch. Biochem. Biophys..

[82] Cuadrado A., Pajares M., Benito C., Jimenez-Villegas J., Escoll M., Fernandez-Gines R., Garcia Yague A.J., Lastra D., Manda G., Rojo A.I. (2020). Can Activation of NRF2 Be a Strategy against COVID-19?. Trends Pharmacol. Sci..

[83] George P.M., Wells A.U., Jenkins R.G. (2020). Pulmonary fibrosis and COVID-19: The potential role for antifibrotic therapy. Lancet Respir. Med..

[84] Varga Z., Flammer A.J., Steiger P., Haberecker M., Andermatt R., Zinkernagel A.S., Mehra M.R., Schuepbach R.A., Ruschitzka F., Moch H. (2020). Endothelial cell infection and endotheliitis in COVID-19. Lancet.

[85] Sbirkov Y., Dzharov V., Todorova K., Hayrabedyan S., Sarafian V. (2022). Endothelial inflammation and dysfunction in COVID-19. Vasa.

[86] Crook H., Raza S., Nowell J., Young M., Edison P. (2021). Long covid-mechanisms, risk factors, and management. BMJ.

[87] Del Rio C., Collins L.F., Malani P. (2020). Long-term Health Consequences of COVID-19. JAMA.

[88] Manoharan Y., Haridas V., Vasanthakumar K.C., Muthu S., Thavoorullah F.F., Shetty P. (2020). Curcumin: A Wonder Drug as a Preventive Measure for COVID-19 Management. Indian. J. Clin. Biochem..

[89] Deng W., Xu Y., Kong Q., Xue J., Yu P., Liu J., Lv Q., Li F., Wei Q., Bao L. (2020). Therapeutic efficacy of Pudilan Xiaoyan Oral Liquid (PDL) for COVID-19 in vitro and in vivo. Signal Transduct. Target. Ther..

[90] Schettig R., Sears T., Klein M., Tan-Lim R., Jr R., Aussems C., Hummel M., Sears R., Poteet Z., Warren D. (2020). COVID-19 Patient with Multifocal Pneumonia and Respiratory Difficulty Resolved Quickly: Possible Antiviral and Anti-Inflammatory Benefits of Quercinex (Nebulized Quercetin-NAC) as Adjuvant. Adv. Infect. Dis..

[91] Rothwell J.A., Urpi-Sarda M., Boto-Ordonez M., Llorach R., Farran-Codina A., Barupal D.K., Neveu V., Manach C., Andres-Lacueva C., Scalbert A. (2016). Systematic analysis of the polyphenol metabolome using the Phenol-Explorer database. Mol. Nutr. Food Res..

[92] Wishart D.S., Guo A., Oler E., Wang F., Anjum A., Peters H., Dizon R., Sayeeda Z., Tian S., Lee B.L. (2022). HMDB 5.0: The Human Metabolome Database for 2022. Nucleic Acids Res..

[93] Wang M., Carver J.J., Phelan V.V., Sanchez L.M., Garg N., Peng Y., Nguyen D.D., Watrous J., Kapono C.A., Luzzatto-Knaan T. (2016). Sharing and community curation of mass spectrometry data with Global Natural Products Social Molecular Networking. Nat. Biotechnol..

[94] Zhang R., Zhu X., Bai H., Ning K. (2019). Network Pharmacology Databases for Traditional Chinese Medicine: Review and Assessment. Front. Pharmacol..

[95] Horby P., Mafham M., Linsell L., Bell J.L., Staplin N., Emberson J.R., Wiselka M., Ustianowski A., Elmahi E., RECOVERY Collaborative Group (2020). Effect of Hydroxychloroquine in Hospitalized Patients with COVID-19. N. Engl. J. Med..

[96] Thomas S., Patel D., Bittel B., Wolski K., Wang Q., Kumar A., Il’Giovine Z.J., Mehra R., McWilliams C., Nissen S.E. (2021). Effect of High-Dose Zinc and Ascorbic Acid Supplementation vs Usual Care on Symptom Length and Reduction Among Ambulatory Patients With SARS-CoV-2 Infection: The COVID A to Z Randomized Clinical Trial. JAMA Netw. Open.

[97] Capasso L., De Masi L., Sirignano C., Maresca V., Basile A., Nebbioso A., Rigano D., Bontempo P. (2025). Epigallocatechin Gallate (EGCG): Pharmacological Properties, Biological Activities and Therapeutic Potential. Molecules.

[98] Zhang Z., Hao M., Zhang X., He Y., Chen X., Taylor E.W., Zhang J. (2023). Potential of green tea EGCG in neutralizing SARS-CoV-2 Omicron variant with greater tropism toward the upper respiratory tract. Trends Food Sci. Technol..

[99] Hayashi A., Terasaka S., Nukada Y., Kameyama A., Yamane M., Shioi R., Iwashita M., Hashizume K., Morita O. (2022). 4″-Sulfation Is the Major Metabolic Pathway of Epigallocatechin-3-gallate in Humans: Characterization of Metabolites, Enzymatic Analysis, and Pharmacokinetic Profiling. J. Agric. Food Chem..

[100] Kiselevsky D.B., Samuilova O.V., Samuilov V.D. (2023). Epigallocatechin Gallate: pH-Dependent Redox Properties and Effect on Respiration, Photosynthesis, and Cell Death in Pea Plants. Biochemistry.

[101] Zhang S., Mao B., Cui S., Zhang Q., Zhao J., Tang X., Chen W. (2024). Absorption, metabolism, bioactivity, and biotransformation of epigallocatechin gallate. Crit. Rev. Food Sci. Nutr..

[102] Esmaeili Z., Shavali Gilani P., Khosravani M., Motamedi M., Maleknejad S., Adabi M., Sadighara P. (2025). Nanotechnology-driven EGCG: Bridging antioxidant and therapeutic roles in metabolic and cancer pathways. Nanomedicine.

[103] Fan Y., Zhou Y., Zhao J., Zhao Y. (2025). Advances in Inhaled Nanoparticle Drug Delivery for Pulmonary Disease Management. FASEB J..

[104] Lin C.C., Hsieh C.Y., Chen L.F., Chen Y.C., Ho T.H., Chang S.C., Chang J.F. (2023). Versatile Effects of GABA Oolong Tea on Improvements in Diastolic Blood Pressure, Alpha Brain Waves, and Quality of Life. Foods.

[105] Lin C.C., Lin H.H., Chang H., Chuang L.T., Hsieh C.Y., Lu S.H., Hung C.F., Chang J.F. (2022). Prophylactic Effects of Purple Shoot Green Tea on Cytokine Immunomodulation through Scavenging Free Radicals and NO in LPS-Stimulated Macrophages. Curr. Issues Mol. Biol..

[106] Rovaldi E., Di Donato V., Paolino G., Bruno M., Medei A., Nisticò S.P., Pellacani G., Kiss N., Azzella G., Banvolgyi A. (2025). Epigallocatechin-Gallate (EGCG): An Essential Molecule for Human Health and Well-Being. Int. J. Mol. Sci..

[107] Hu J., Webster D., Cao J., Shao A. (2018). The safety of green tea and green tea extract consumption in adults—Results of a systematic review. Regul. Toxicol. Pharmacol..

[108] Grajecki D., Ogica A., Boenisch O., Hubener P., Kluge S. (2022). Green tea extract-associated acute liver injury: Case report and review. Clin. Liver Dis..

[109] Younes M., Aggett P., Aguilar F., Crebelli R., Dusemund B., Filipic M., Frutos M.J., Galtier P., Gott D., EFSA Panel on Food Additives and Nutrient Sources added to Food (ANS) (2018). Scientific opinion on the safety of green tea catechins. EFSA J..

[110] Yan R., Cao Y. (2025). The Safety and Efficacy of Dietary Epigallocatechin Gallate Supplementation for the Management of Obesity and Non-Alcoholic Fatty Liver Disease: Recent Updates. Biomedicines.

[111] Au-Doung P.L.W., Mak K.K.W., To K.K.W. (2025). Green tea supplementation in adults with obesity: A systematic review of clinical studies. Discov. Food.

[112] Furushima D., Nishimura T., Takuma N., Iketani R., Mizuno T., Matsui Y., Yamaguchi T., Nakashima Y., Yamamoto S., Hibi M. (2019). Prevention of Acute Upper Respiratory Infections by Consumption of Catechins in Healthcare Workers: A Randomized, Placebo-Controlled Trial. Nutrients.

[113] Umeda M., Tominaga T., Kozuma K., Kitazawa H., Furushima D., Hibi M., Yamada H. (2021). Preventive effects of tea and tea catechins against influenza and acute upper respiratory tract infections: A systematic review and meta-analysis. Eur. J. Nutr..

[114] Carr A.C., Rowe S. (2020). The Emerging Role of Vitamin C in the Prevention and Treatment of COVID-19. Nutrients.

[115] Bui L., Zhu Z., Hawkins S., Cortez-Resendiz A., Bellon A. (2021). Vitamin D regulation of the immune system and its implications for COVID-19: A mini review. SAGE Open Med..

[116] Liu C.-Y., Hsieh C.-Y., Chang S.-H., Wang S.-C., Lin C.-C. (2023). Effects of a nutraceutical combining green tea extract, vitamin C, D, and zinc in patients with post-COVID conditions. J. Food Sci. Nutr. Ther..

[117] Williamson G., Kerimi A. (2020). Testing of natural products in clinical trials targeting the SARS-CoV-2 (COVID-19) viral spike protein-angiotensin converting enzyme-2 (ACE2) interaction. Biochem. Pharmacol..

[118] Liu J., Bodnar B.H., Meng F., Khan A.I., Wang X., Saribas S., Wang T., Lohani S.C., Wang P., Wei Z. (2021). Epigallocatechin gallate from green tea effectively blocks infection of SARS-CoV-2 and new variants by inhibiting spike binding to ACE2 receptor. Cell Biosci..

[119] Hurst B.L., Dickinson D., Hsu S. (2021). Epigallocatechin-3-Gallate (EGCG) Inhibits SARS-CoV-2 Infection in Primate Epithelial Cells: (A Short Communication). Microbiol. Infect. Dis..

[120] Guo X., Liu H., Hou R., Chen G., Xiao H., Liu L., Ciftci O.N., Liu L. (2024). Design strategies of polysaccharide, protein and lipid-based nano-delivery systems in improving the bioavailability of polyphenols and regulating gut homeostasis. Int. J. Biol. Macromol..

[121] El-Saadony M.T., Yang T., Saad A.M., Alkafaas S.S., Elkafas S.S., Eldeeb G.S., Mohammed D.M., Salem H.M., Korma S.A., Loutfy S.A. (2024). Polyphenols: Chemistry, bioavailability, bioactivity, nutritional aspects and human health benefits: A review. Int. J. Biol. Macromol..

[122] Lai Y.J., Liu S.H., Manachevakul S., Lee T.A., Kuo C.T., Bello D. (2023). Biomarkers in long COVID-19: A systematic review. Front. Med..

[123] Morelli T., Purcell M., Rodrigues P., Roberts C., Cox O., Lee P.H., Thorne K., Allen A., Cazaly A., Nuttall J. (2025). Understanding Infection, Viral Exacerbation and Respiratory Symptoms at Admission-Longitudinal (UNIVERSAL) study: A prospective observational cohort study protocol. BMJ Open.

[124] Oronsky B., Larson C., Hammond T.C., Oronsky A., Kesari S., Lybeck M., Reid T.R. (2023). A Review of Persistent Post-COVID Syndrome (PPCS). Clin. Rev. Allergy Immunol..

[125] Dutta D., Liu J., Xiong H. (2022). NLRP3 inflammasome activation and SARS-CoV-2-mediated hyperinflammation, cytokine storm and neurological syndromes. Int. J. Physiol. Pathophysiol. Pharmacol..

