# Supplementary material for: Tea Polyphenols in the COVID-19 Era: Mechanistic Insights and Translational Challenges
Source: Curr Issues Mol Biol. 2026 Apr 5;48(4):379. doi: 10.3390/cimb48040379 (PMC13115506; doi:10.3390/cimb48040379)
Supplement: Supplementary file 1 [file cimb-48-00379-s001.zip › cimb-4199910-supplementary.pdf]

Supplementary Material

Table S1. Molecular docking and inhibitory profiles of various dietary polyphenols against SARS-CoV-2 targets.

Viral Target	Phytochemical / Source	Key Structural Motifs	Binding / Inhibitory Profiles	References
ACE2 / Spike	Curcumin	Phenolic hydroxyl groups	Interacts with Q493, T501, Y505, Y489, and Q498 via strong H-bonds.	[37]
3CLpro (Mpro)	Rutin	Flavonol glycoside	Strongest predicted binding to the active site (PDB: 6Y84) among 33 compounds.	[67]
3CLpro / Spike	Tangeretin, Naringenin (Citrus & Galangal)	Polymethoxylated / Flavanone	High binding affinity; performed better than lopinavir and nafamostat in silico.	[38]
PLpro / 3CLpro	Chalcones (Angelica keiskei)	Open-chain flavonoids	Noncompetitive mechanism against PLpro; competitive against 3CLpro.	[71]
3CLpro / PLpro	Kaempferol, Isoliquiritigenin	Hydroxylated flavonoids	Synergistic dual inhibition observed in vitro.	[72]
Spike (RBD)	Luteolin, Quercetin	Flavone/Flavonol	Blocks viral entry by binding to the spike protein.	[76, 77]
Spike-ACE2	Emodin (Rheum officinale)	Anthraquinone	IC_{50} in the 1–10 μg/mL range; inhibits spike interaction.	[78, 79]
Multi-target	Toremifene + Emodin	Combined Scaffolds	Synergistic antiviral effects predicted via systems-pharmacology.	[80]
RdRp	Fenoterol, Baicalin, Xanthones	Flavone / Xanthone scaffold	Identified as potent RdRp inhibitors via computational screening.	[90]

RdRp	EGCG, Myricetin, Quercetagetin	Hydroxylated flavonols	Strong binding affinity toward both SARS-CoV and SARS-CoV-2 RdRp.	[91]
